# Elucidating the effect of tomato leaf surface microstructure on *Botrytis cinerea* using synthetic systems

**DOI:** 10.3389/fpls.2022.1023502

**Published:** 2022-10-27

**Authors:** Helen Rombach, Haguy Alon, Orr H. Shapiro, Yigal Elad, Maya Kleiman

**Affiliations:** ^1^ Department of Agriculture and Horticulture, Humboldt University Zu Berlin, Berlin, Germany; ^2^ Institute of Plant Sciences, Department of Vegetables and Field Crops, Agricultural Research Organization (Volcani Center), Rishon Lezion, Israel; ^3^ Inter-Faculty Graduate Biotechnology Program, the Hebrew University of Jerusalem, Rehovot, Israel; ^4^ Institute of Postharvest and Food Sciences, Department of Food Sciences, Agricultural Research Organization (Volcani Center), Rishon Lezion, Israel; ^5^ Institute of Plant Protection, Department of Plant Pathology and Weed Research, Agricultural Research Organization (Volcani Center), Rishon Lezion, Israel; ^6^ Agro-Nano Technology and Advanced Materials Center, Agricultural Research Organization (Volcani Center), Rishon Lezion, Israel

**Keywords:** biomimetics, gray mold, microstructure, surface, thigmo-response, tomato

## Abstract

For some pathogenic fungi, sensing surface topography is part of their infection strategy. Their directional growth and transformation to a new developmental stage is influenced by contact with topographic features, which is referred to as thigmo-response, the exact functionality of which is not fully understood. Research on thigmo-responses is often performed on biomimetically patterned surfaces (BPS). Polydimethylsiloxane (PDMS) is especially suitable for fabrication of BPS. Here, we used synthetic BPS surfaces, mimicking tomato leaf surface, made from PDMS with the pathogenic fungus *Botrytis cinerea* to study the influence of structural features of the leaf surface on the fungus behavior. As a control, a PDMS surface without microstructure was fabricated to maintain the same chemical properties. Pre-penetration processes of *B. cinerea*, including the distribution of conidia on the surface, germination, and germ tube growth were observed on both leaf-patterned and flat PDMS. Microstructure affected the location of immediate attachment of conidia. Additionally, the microstructure of the plant host stimulated the development of germ tube in *B. cinerea*, at a higher rate than that observed on flat surface, suggesting that microstructure plays a role in fungus attachment and development.

## 1 Introduction

The interaction between fungal pathogens and plant hosts often begins when surface contact is established (Zelinger et al., 2006). Successful pathogen infection requires a sophisticated mechanism to overcome physical barriers and chemical defenses on the host surface. The fungal conidia attach to the surface in a two-step process: Immediate attachment that happens within the first seconds of binding, and adhesion that occurs after a few hours of incubation under conditions that will allow germination (Doss et al., 1993), where germination of conidia is the transformation of a dormant cell into a growing hypha. This process marks a fundamental step in fungal development. It involves the breaking of dormancy by external signals, followed by the formation of a germ tube (d’Enfert, 1997; Barhoom and Sharon, 2004).

One well studied system in the context of plant-pathogen interaction is tomato-Botrytis cinerea (Fillinger and Elad, 2016). Tomato is one of the most important commercial crops in the world, with a production value estimated at more than $50 billion (Vincent et al., 2013). Tomato leaves are an example for densely glandular-hairy leaves with hydrophobic, waxy cuticle, produced by epidermal cells, as a protective cover. The cuticle prevents water loss of the leaf and effectively reduce pathogen entry due to waxy secretion (Yeats and Rose, 2013). Specially, the cuticle is an important barrier against pathogen attacks (Yang et al., 2018).

B. cinerea is a pathogenic fungus, which poses a significant threat to tomato growth (O’Neill et al., 1997). B. cinerea attacks more than 1400 plant hosts worldwide (Elad et al., 2016; Veloso and van Kan, 2018). The fungus is characterized by gray-brown conidia, which are usually transmitted by air movement (Williamson et al., 2007) and the disease is commonly known as gray mold. Difficulties in controlling B. cinerea result from the ability of the fungus to infect all parts of the plant at almost all developmental stages and agricultural products during transport and storage. Disease control methods being explored and applied are breeding for host resistance (Soltis et al., 2019), cultural practices (Elad, 2016), biological (Elad, 2000; Calvo-Garrido et al., 2013) and chemical (Elad et al., 1992; Korolev et al., 2011; Mouekouba et al., 2013) control. However, each one of the methods poses a set of problems. B. cinerea often penetrates through wounds (Williamson et al., 2007), but direct penetration of germ tubes via natural openings (Verhoeff, 1970) or through the cuticle into undamaged tissue has also been observed (Verhoeff, 1970; Elad, 1988; Elad, 1989).

Previous studies showed that binding of B. cinerea conidia to the plant surface, germination and germ tube growth are complex processes that depend on a combination of factors. These elements include both physical signals, such as the hardness, topography and hydrophobicity of the plant surface, and chemical signals including cuticular waxes, cutin monomers and various nutrients (Doss et al., 1993; Mendgen et al., 1996; Tucker and Talbot, 2001; Doehlemann et al., 2006; Yang et al., 2018). There has been a strong research effort to identify chemical components of the cuticular waxes that induce pre-penetration processes and molecular pathways of *B. cinerea* infection (Leroch et al., 2013; Silva-Moreno et al., 2016).

In other pathogenic fungi, a mechanism of mechanochemical sensing has been reported. While the exact functionality of this mechanism is not fully understood (Almeida and Brand, 2017), it is, however, clear that sensing surface topography (thigmo-based response) plays a central role in the infection strategy. Host leaf surface topography has been shown to be important in recognition and attachment of conidia (Nicholson and Epstein, 1991; Mercure et al., 1994). Specifically, their directional growth and transformation to a new development stage is influenced by contact with topographic features, such as cell interfaces and stomata (Hoch et al., 1987). For example, conidia of the pathogen *Stagonospora nodorum* bound more frequently to wheat leaf surface than to barley leaves and recognition of dimensional properties, such as ridges, furrows and trichomes was suggested to contribute to this selection (Zelinger et al., 2006). However, the influence of surface microstructure in the attachment of *B. cinerea* conidia to surface has not been addressed.

A significant problem in studying plant surface-microorganism interactions is the variations of the surface microstructure of plant leaves. The surface microstructure of leaves varies with species, cultivar, plant and location on the plant, and is influenced by growing conditions and maturity stage. Therefore, replication of experiments and interpretations of variations as a function of experimental parameters is difficult.

A solution to this problem can arise from the field of biomimetics. In material science, biomimetic approaches have enabled the systematic study of nature inspired nano-, micro- and macroscopic structures (Wu et al., 2016). Perhaps most famous are biomimetic surfaces reproducing the water repellency of lotus leaves. These superhydrophobic surfaces have been utilized in technological applications such as self-cleaning, self-repairing interfaces (Zorba et al., 2008). Specifically, development of biomimetically patterned surfaces (BPS) that faithfully and reproducibly replicate the microstructure topography of plant leaves can solve the mentioned problems. Polydimethylsiloxane (PDMS) is particularly suitable for replicating the surface structure of plant leaves (Zhang et al., 2014; Wu et al., 2016). BPS provide means to precisely replicate experiments and allow interpretation of results without influence of natural variations. Beyond providing multiple identical copies of the leaf structure, BPS also provide a mean to examine the role of surface topography in surface attachment of microorganism. This is because the use of BPS allows to exclude chemical components that impact leaf-microorganism interaction on the natural leaf (Zhang et al., 2014).

Here we aim to highlight the effect of leaf surface microstructure on leaf-pathogenic microorganism interaction using conidia of *B. cinerea* at a pre-penetration stage and tomato leaves as our model system. We tested the influences of leaf surface microstructure on the distribution of conidia, germination of conidia and germ tube growth. This study focuses on the topographical signals that influence *B. cinerea* conidia distribution and germination. We tested whether the leaf microstructure is stimulating the induction of germination by comparing the reaction to PDMS based BPS to that of a PDMS flat surface. Comparing the germination on natural leaf and glass, as done in previous studies, does not allow to exclude the influence of potential chemical stimuli from the plant leaf and is therefore unsuited to study thigmo-responses of fungi. Comparing germination on PDMS that replicate the microstructure of tomato leaves with germination on PDMS without microstructure, can hence help us to gain insight on the influence that the microstructure has on the germination process of *B. cinerea.*

## 2 Materials and methods

### 2.1 *Botrytis cinerea* culture

*B. cinerea* isolate Bc-16 (Elad and Yunis, 1993) was grown on potato dextrose agar (PDA, Difco, NJ, USA) medium (composed of 2.2% PDA and containing 250 mg L^-1^ chloramphenicol (Sigma Aldrich, St. Louis, MO, USA)) at 19°C for 10 days. The conidia were removed from the growth medium surface by a wash with sterilized distilled water containing 0.2% glucose and 0.2% KH_2_PO_4_ (both from Sigma-Aldrich, St. Louis, MO, USA) and then filtered using four layers of sterilized gauze to remove hypae fragments. Conidia were counted using a Hemocytometer.

### 2.2 Flat PDMS surface fabrication

Sylgard 184 polymer kit (Dow Corning, Midland, MI, USA) was used. Prepolymer and curing agent were well mixed at a 10/1 w/w ratio of polymer/curing agent, respectively, and then kept under vacuum for 1h to remove air bubbles. The solution was poured into a Petri dish to form a thin layer of approximately 0.2 mm thickness.

### 2.3 Biomimetically patterned surfaces (BPS) fabrication

**Figure 1:**
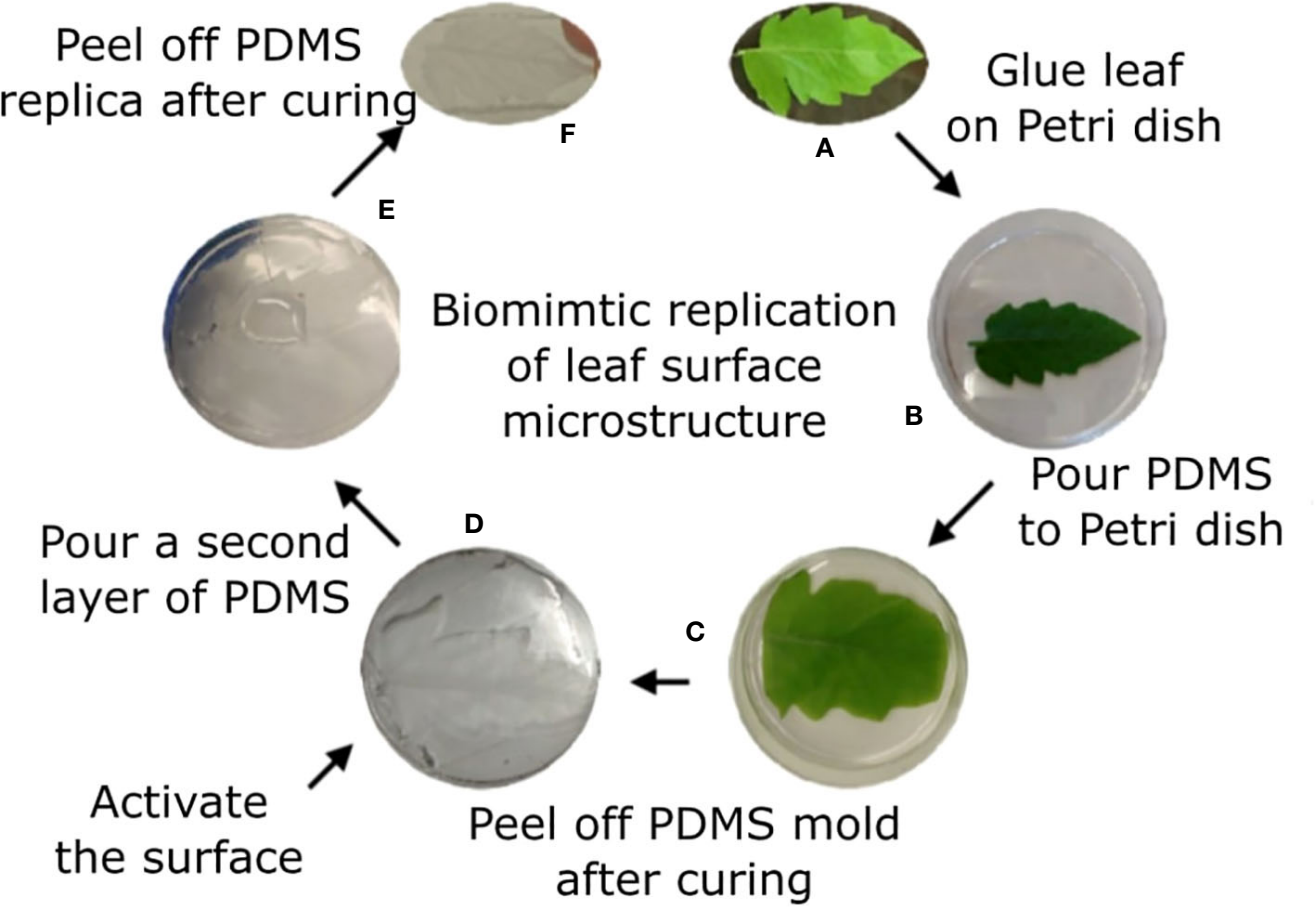
Tomato leaf replication process. Tomato leaf **(A)** is glued on a Petri dish with the abaxial side facing up **(B)**. Liquid PDMS is poured on top of the leaf **(C)** and cured overnight. The leaf is peeled off the surface, generating a negative replication of the leaf surface, and the surface is activated using plasma **(D)**. A second layer of PDMS is poured on the negative replica **(E)** and cured for 30 min. The two layers are separated, and the positive replica is now visible on the later polymer **(F)**.

Sylgard 184 polymer kit was used. Prepolymer and curing agent were well mixed at a 10/1 w/w ratio of polymer/curing agent, respectively and then kept under vacuum for 1h to remove air bubbles. The natural leaf (4th leaf from a M82 one month old tomato plant) was taped to a Petri dish with the abaxial surface facing up. The polymer solution was poured on top of the leaf. Vacuum was applied for 2h to assure full coverage of the surface microstructure. The covered leaf was kept at room temperature overnight. The leaf was then peeled off the cured polymer, leaving the PDMS template of the leaf surface microstructure mirror image (negative replica). To avoid attachment of the positive replica onto this negative replica, a functionalization process was performed. A BD-20AC laboratory CORONA treater (Electro-Technic Products, Chicago, IL, USA) was used for a few seconds to activate the surface of the negative replica. The negative replica was then immediately placed in a desiccator with 100 µl of Trichloro(1H,1H,2H,2H-perfluorooctyl)silane (Sigma Aldrich, St. Louis, MO, USA) for 3h. The negative replica was then placed in a Petri dish and liquid PDMS (10:1, as described previously) was poured on top, creating a thin layer of polymer. This construct was vacuumed for 1h and a microscope glass slide was placed on top of it to assure the resulted template is thin and flat. Curing occurred at 65°C for 30 min. The negative replica was then carefully removed from the newly formed polymer layer to achieve the replication of the leaf surface microstructure (positive replica). The process is described in **Figure 1**.

### 2.4 Scanning electron microscopy (SEM)

Analysis of PDMS replica surface topography was performed using a JCM-6000PLUS NeoScope Benchtop electron scanning microscopy (Jeol, Tokyo, Japan). The PDMS replica was cut into small pieces of 4x4 mm and coated with gold using a sputter coater (E-1010, Hitachi, Japan). Images were captured at a magnification of 100x at 15kV acceleration voltage. Images of the natural leaf were also taken under the same conditions after coating. Before coating the leaves were dehydrated using incubation at RT in elevated ethanol concentrations (70, 80, 90 and 100%) for 1h each, followed by Critical Point Drying (CPD) using K850 Quorum Critical Point Dryer (Quorum, Laughton, UK).

### 2.5 Live imaging

Microscope imaging was performed using a NIKON eclipse T*i* microscope (Nikon, Japan) equipped with a ProScan motorized XY stage (Prior Scientific, MA, USA) with a temperature-controlled incubator (LAUDA ECO RE 415, Korea). Bright field illumination provided by a cool LED pE-100A (Cool LED, UK). Imaging was performed using an ANDOR zyla 5.5 MP ScMOS camera (China) and processed using the NIS elements AR 4.6 (64 bit) software package. 3 mm diameter circles of both PDMS replica and the flat PDMS were cut using a puncher. Three samples of each structure were put in the same chamber of a 24 wells vision plate. 10 µl of *B. cinerea* conidia suspension at 10^5^ conidia/ml was added into the chamber. The drop of 10 µl was applied on top of the surface, at no particular location. Since there were several repeats, and since the drop is significantly larger than all microscopic features, we assumed a good coverage of microstructural features by the conidia. Images were captured every 15 minutes for 18 hr at six locations, three locations on each surface type.

### 2.6 Image analysis

The captured area of the image was 1715x1447 µm^2^. Images were analyzed using ImageJ software. A grid of 82x82 µm^2^ squares was added to the image. For conidia distribution calculation, an area of 72 (12x6) squares in high focus was analyzed. Conidia in each square of the grid were counted giving number of conidia per square. For structural features analysis, areas of structural features were measured by the ‘freehand selection’ tool. Calculation of total leaf cell area was done by measuring three cells in the relevant image and multiplying the average size by the total number of cells in the image frame. Similarly, cell interface length and width were measured for three cells and the average was multiplied by the total number of cells. For germination calculation, an area of 25 (5x5) squares was selected. The total amount of conidia at t=0 was counted. Germination was observed every hour for 18h by counting the number of conidia that developed germ tubes. Germ tube length of each germinating conidia was measured at 3 time points (4, 8, and 12h) manually using the ‘free line’ tool.

### 2.7 Statistical analysis

#### 2.7.1 Conidia distribution

Conidia were counted in every square of the grid. The average number of conidia per square was obtained. For each square, the number of conidia relative to the average was calculated by dividing the number of conidia in the square by the average. As a result, each square was given a number between 0 and 3.9. This range (0-3.9) was divided to sections of 0.3 size. The process was performed on 3 separated images from each type. The average number of squares in each section of the same surface type was calculated and the distribution was graphed. Standard deviations for each section were also added. The two distributions were subjected to Shapiro-Wilk test using the online tool to assess their resemblance to a normal distribution.

#### 2.7.2 Conidia germination rate

To test for the difference in germination rate between the two surfaces, we counted the entire population of conidia in the frame and the number of germinated conidia in each time point. We first normalized the conidia population of each condition. This was done by subtraction of the average conidia population size on each surface type from the number of conidia counted on a specific surface. We then calculated the germination proportions (p), that is the part of germinated conidia out of the entire population (a number between 0 and 1). We performed a Logit Transformation (LT) for each proportion: $LT=\;\text{ln}\left(\frac{P}{1-p}\right)$ . We then performed a linear regression for all repeats of the same conditions between the normalized population size and the LT of germinated conidia proportion to clear the population size effect from the conidia tendency to germinate. At this point we “cleaned” the LT by subtracting from it the y axis intersect from the linear regression times the normalized population size. That is, if the linear regression resulted in an equation y=a*x+b, then our “clean” LT was equal to: LT-b*[P], where [P] is the normalized population size. We then transformed the clean LT back to proportion by: $clean\;p=\;\frac{1}{1+e^{-LT\;clean}}$ . This new clean proportion was then multiplied by the population size to generate the “true” normalized number of germinated conidia in this image. For each time point the normalized number of germinated conidia from all images under the same condition (flat or patterned surface) were added together and divided by the total number of conidia from that condition to generate the total proportion of germinating conidia under this condition. The two arrays (proportion of germination on flat surface and proportion of germination on patterned surface) in all different time points were subjected to a two sample binomial test to achieve the *p*-value.

#### 2.7.3 Germ tube length

To test for significant difference in germ tube length between the two groups (the conidia germinating on flat surface vs. the conidia germinating on patterned surface) we measured the germ tube length of each of the germinating conidia on each surface at three time points (4, 8, and 12h). For each time point, we divided each vector containing the different germ tube lengths on each surface type into bins, where the number of bins ranged between 5 and 100. We then calculated the KL divergence between the vector representing the different germ tube length on flat surface and the one representing the different germ tube length on patterned surface. At this point, we randomly distributed the values representing the germ tube lengths between the two vectors and calculated the KL divergence again. This step was repeated 1000 times and the percentage of times in which the value of the KL divergence was higher than the original value was returned. The assumption underlining this process was that if this percentage is lower than 5% then the difference in germ tube length between the two groups is statistically significant.

## 3 Results

### 3.1 Synthetic replication of tomato leaves

**Figure 2:**
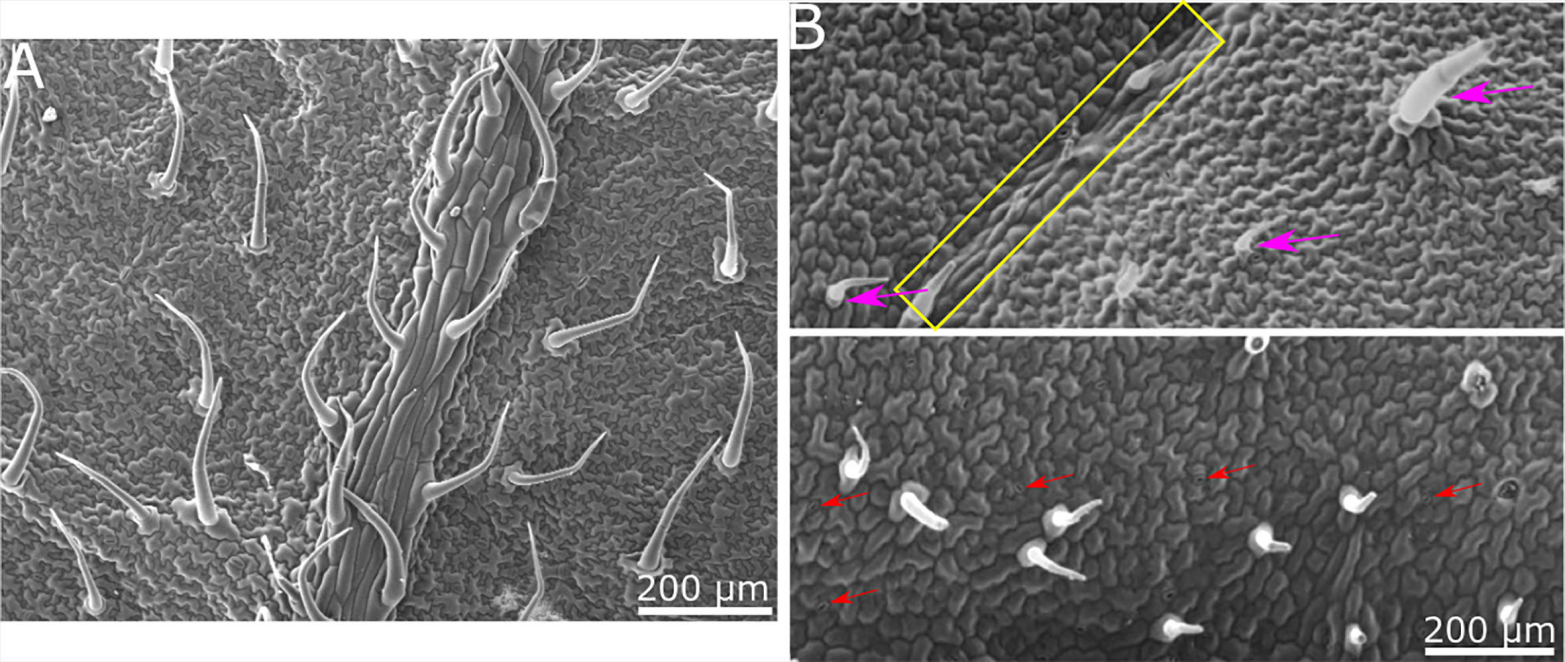
SEM images of tomato leaf and replica made of PDMS. **(A)** SEM micrograph of tomato leaf. **(B)** SEM micrograph of PDMS replica. All relevant structural features of the tomato leaf surface are visible in the replica. Secondary vein is indicated by a yellow rectangle in the upper image. Trichomes are marked with purple arrows in the upper image. Stomata are indicated with red arrows in the lower image. Epidermis cell structure is also replicated.

Our goal was to determine whether there is a physical/structural element in the response of *B. cinerea* upon landing on a surface. We hence wished to separate the physical/structural element of the surface from all other properties. To that end, we generated a synthetic replica of the abaxial tomato leaf surface microstructure using the biocompatible, silicone-based polymer PDMS (**Figure 1**). The replicated surface was examined using Scanning Electron Microscopy (SEM) to ascertain the level to which the leaf microstructure has been reproduced (**Figure 2**). The abaxial surface of tomato leaves has several characteristic structural features such as central vein (midrip), secondary veins, stomata and hairs (trichomes), ranging in scale from 10s to 100s microns. We could find all relevant structural features of the natural leaf in the replica (**Figure 2**), although, some leaf trichome breakage was noted. This phenomenon was previously observed in a study replicating bean leaves to understand trichome role in the capture of bed bugs by these leaves (Szyndler et al., 2013). However, since the replication of the base of the trichomes was always present on the surface, and many trichomes were replicated in full, we can say that our replica represents a replication of the tomato leaf surface structure on a synthetic, inert surface. As such, it can assist in isolating the structural influence of the surface on *B. cinerea* conidia behavior.

### 3.2 Distribution of conidia on patterned and non-patterned surfaces

**Figure 3:**
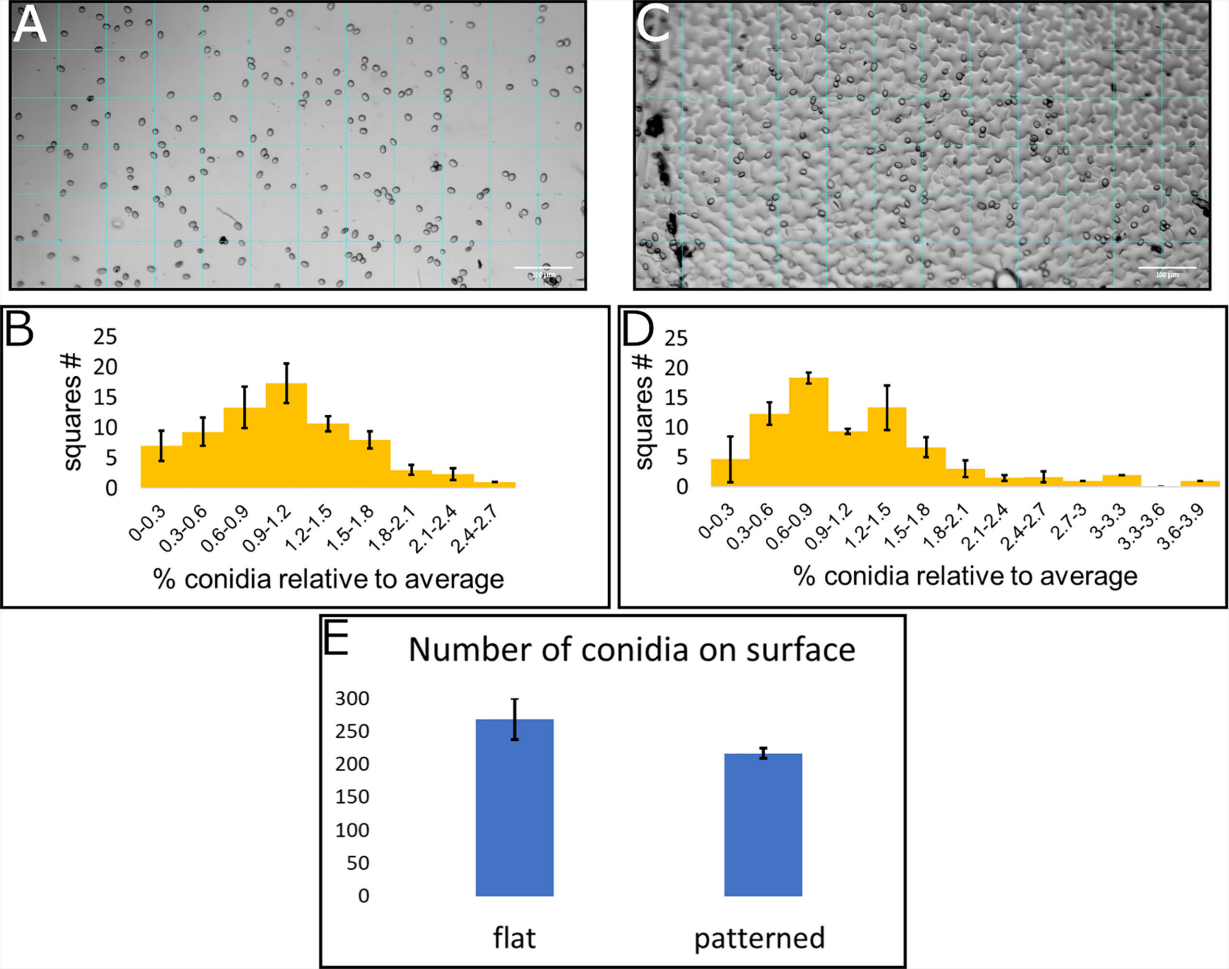
Conidia distribution on flat and patterned surfaces. 10μl of *B*. *cinerea* conidia suspension (10^5^ conidia/ml) was applied on a flat surface **(A)** and a tomato leaf replica **(C)**, both made from PDMS. A grid of 72 squares was applied to the microscope images (**A** for flat surface and **C** for patterned surface). The number of conidia was counted in each square and a histogram of number of squares with different amounts of conidia compared to the average was generated (**B** for flat surface and **D** for patterned surface). **(E)** shows the total number of conidia counted on these surfaces. Three repeats of each surface type were performed and insert into the histogram.

To assess the conidia distribution upon the surface, and how it correlates with structural features, we used both the replication of the leaf surface microstructure made from PDMS and a flat surface also made from PDMS. Both surfaces were chemically identical and differed only in their structure. Conidia suspension was applied to each of the surfaces and an image was taken using light microscopy. A grid of 72 squares with a size of 82µmx82µm was applied to every image (**Figures 3A, C** show representative images). The image clearly shows the conidia upon the surface as well as the structural features. The conidia in every square were counted, the average number per square was calculated and the ratio between the number of conidia on the square and the average number of conidia per square was calculated. The distribution of conidia, as number of squares containing specific range of conidia compared to average is presented (**Figures 3B, D**). The number of conidia on each of the inspected surfaces was similar and around 200 conidia for the whole surface examined (**Figure 3E**). Three surfaces from each type were analyzed. Under Shapiro-Wilk test, none of the distributions was normal, however, the distribution of conidia on the flat surface resembles a normal distribution more than the conidia distribution on patterned surface (compare **Figures 3B, D**). This is also confirmed by the skewness and kurtosis which were lower for the distribution of conidia on the flat surface (skewness of 0.51 and 1.02 and kurtosis of -0.02 and 1.51 for conidia distribution on flat and patterned surfaces, respectively) and the *p*-value which was higher for distribution on a flat surface than the one on patterned surface by four orders of magnitude. The deviation from normal distribution on the flat surface was likely caused by the apparent aggregation of conidia that was visible on the surface, creating the slightly longer tail at the high number of conidia per square compared to the low number of conidia per square (**Figure 3B**). The distribution on the structured surface, on the other hand, did not resemble a normal distribution at all. For example, we can see a reduction in the graph where the average is, in contrast to normal distribution where the average is the highest point of the graph (**Figure 3D**). Additionally, the tail with the high number of conidia was much longer than that of the flat surface (**Figures 3D** compared to **3B**). These results confirm that the structure plays a role in conidia distribution on the surface. It seems that there is a higher tendency for conidia clustering on the patterned surface that mimics tomato leaf surface structure than on the flat surface.

To further characterize the effect of structure on conidia distribution we counted the number of conidia on the different structural features. Leaf surface structure is characterized by several structural features such as: veins, stomata, trichomes and cell interfaces. The analysis of conidia distribution regarding their location on a specific feature of the artificial leaf surface showed a strong accumulation of conidia at cell boundaries (**Table 1**). Almost 80% of the conidia were located on the interface between cells, even though they represent only about 11% of the whole surface. 80% of the surface is characterized as cell center, but only ~11% of conidia were located on this area. Additionally, it was observed that 4% of conidia were located on stomata, even though they constitute only 2% of the surface (**Table 1**). The rest of the conidia were located on other areas. Overall, the results confirm that the distribution of conidia is indeed influenced by microstructural features of the leaf surface.

**Table 1:** Conidia distribution on microstructural features.

	Area (%)	Conidia (%)
Cell center	80.0	11.3
Cell interface	11.0	79.8
Stomata	2.0	4.5
Other (veins, trichomes, etc.)	7.0	4.4

The images used in **Figure 3** were further analyzed and the total number of conidia in all repeats of the patterned surface were counted. The area of each structural feature was calculated and the percentage out of the total area is presented. The percentage of conidia on each structural feature is also presented. A clear preference for cell interface and some preference for stomata is detected.

### 3.3 Germination on patterned and smooth surfaces

**Figure 4:**
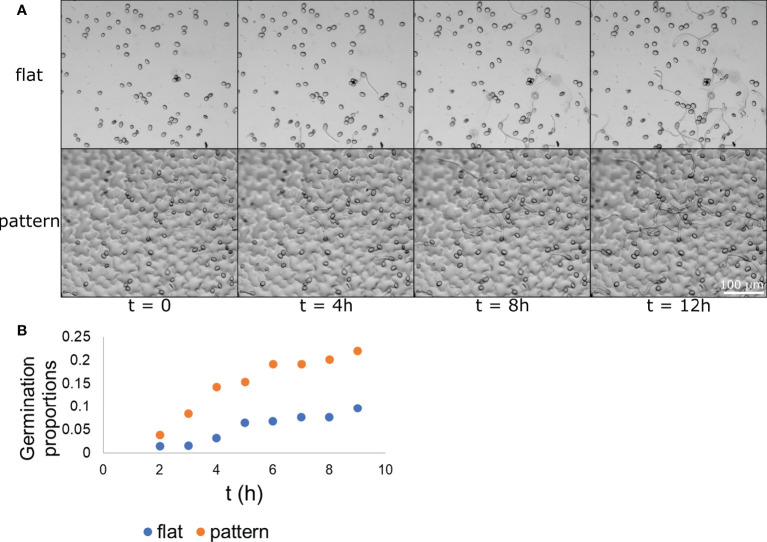
Conidia germination on flat and patterned surfaces. *B. cinerea* conidia were applied to both a flat (A–top) and a patterned (A-bottom) surface. The conidia germination process upon the surface was captured hourly and images from times 0, 4, 8, and 12 h are presented in **(A)** The conidia and their germ tube are clearly visible. The proportion of germinating conidia out of the entire spore population at different time points on each of the surface types was calculated and is presented in **(B)** The difference between the two populations is statistically significant starting from the 3 h time point with *p*<0.0025. n=3.

Our next step was to assess whether the physical effect surface microstructure had on conidia distribution also resulted in a biological effect on conidia activity. To this end, we applied the conidia on the two surface types (flat and patterned) and followed them over time. The conidia were applied with glucose and phosphate (to induce germination) and images of the same location were taken hourly over 18 hours. Representative images of germinating conidia over time on both surface types are presented in **Figure 4A**. At each time point, the number of germinating conidia was counted on each of the surfaces and the proportion of the germinating conidia (their percentage within the total conidia population) was calculated. We first observed germ tube emerging from conidia after one hour, however, the majority of conidia started showing a germ tube after 3-5 hours. After 5 hours the rate of new germination decreased. The proportion of germinated conidia was then normalized to the total conidia population in each one of the surface types separately. It should be noted that this normalization, which was performed using a linear regression, resulted in a negative slope for both surface types. This suggests that the conidia tend to germinate more in smaller populations or, in other words, the presence of other conidia nearby may inhibit germination. Such self inhibition is a known phenomenon (Kritzman et al., 1980). The population size that were examined were between 57 and 88 conidia, meaning, about a 50% change in population size. The normalized proportion of germinating conidia over time was graphed for each of the populations and the result is presented in **Figure 4B**. A binomial test was performed for each one of the time point to test for statistical significance and other than the first time point, for which the *p*-value was 0.1147, all other time points had a *p*-value lower than 0.0025, suggesting a statistical significance between the populations germinating on flat surface and those germinating on patterned surfaces. The maximum germination proportion on the patterned surface, at 9 hour was more than 0.22. This is more than twice the maximum germination proportion on the flat surface, at 9 hours, which was less than 0.1. This result shows that *B. cinerea* conidia “preferred” to germinate on the patterned surface rather than the flat surface. Given the fact that both surfaces were chemically identical as they were both made from PDMS, this result suggests that microstructure plays a role in conidia germination, increasing their tendency to germinate.

Next, we wanted to test whether the growth pattern of the germ tube is influenced by the surface microstructure. In addition to the images taken hourly (example of which is shown in **Figure 4**) we also took videos for 18 h, showing the germination process (**Supplementary Videos 1 and 2**). In these videos, it seemed that some germ tubes directed their growth according to topographic features and elongated along depressions between cells. It was also observed that the growth stagnated before proceeding with a slightly different orientation. On some occasions, the germ tube divided in the area where the stagnation took place. Additionally, on patterned PDMS, swelling of the tip of the germ tube, that are characteristic for appressoria formation were observed after eight hours. Short stagnations and division could also be observed on flat PDMS. Swelling of the germ tube for appressoria formation started later on flat PDMS and was mainly visible after 12 h. It was expected that the more frequent growth stagnation on patterned PDMS would be reflected in a reduced germ tube length compared to flat PDMS. Therefore, the length of the germ tubes was measured and compared. The results of the average and standard deviation of the measurement of germ tube length after 4, 8 and 12 h on patterned and flat PDMS are listed in the top part of **Table 2**. Germ tube length is similar between the two surfaces after 4 and 8 h and becomes slightly higher in the flat surface compared to the patterned surface after 12 h. However, the standard deviation is extremely high in both surfaces indicating a very high variability in germ tube length. We calculated the KL divergence of the two distributions. This gave us a measure as to how different one distribution is from the other. To test for the statistical significance, we randomly distributed the values, to calculate the KL divergence between 1000 random distributions to check how many show a higher value of difference. If most (95% or more) random distributions are less different from one another than the original set, then we can say that there is a significant statistical difference between the two distributions. In this case, we found no statistically significant difference between the two distributions at any of the time points.

**Table 2:** Germ tube length over time.

T	4h		8h		12h	
	flat	patterned	flat	patterned	flat	patterned
Ave	31.00	30.57	82.47	81.73	116.11	105.24
STD	21.45	17.38	42.81	34.88	58.46	46.30

Effect of surface structure and time of incubation on germ tube length (µm). Showing the average length (Ave) and the Standard deviation (STD).

## 4 Discussion

In this study, we aimed at showing that leaf microstructure plays a role in leaf-microorganism interaction of *B. cinerea* on tomato leaf. We wanted to see whether we could observe a thigmo-based response - a critical mechanism for fungi to experience their surroundings and to induce a change to new development stages, also known as the fungal sense of touch (Almeida and Brand, 2017). As our model system we used Botrytis *spp.* that can be found wherever their host crops grow (Elad et al., 2016) ranging from cool temperate zones of Alaska (Anderson, 1924) to subtropical areas like Israel (O’Neill et al., 1997; Shtienberg et al., 1998; Mehari et al., 2015). The fungus attacks agronomically important plants, such as grape vine, strawberries, tomato, cucumber and cut roses (Droby and Lichter, 2007; Elad et al., 2016). Leaf infection by B. cinerea is an important site for gray mold initiation in tomato plants (Shtienberg et al., 1998) and in other crops (Elad, 1990; Shpialter et al., 2009). Hence, leaf surface, where the initial interaction between the fungus and the plant occurs, was the center of the study presented here. We tested whether processes during pre-penetration stage (attachment, germination, germ tube growth) of the fungus are guided through contact-sensing.

To address this question we used BPS. These are surfaces imitating topographical features of the host plant microstructure, while excluding chemical parameters. Leaf surface is one of the most common surfaces used as a target for replication (Bhushan et al., 2009; Koch et al., 2009; Barthlott et al., 2017; Soffe et al., 2019). Traditional fabrication methods of leaf-inspired structures included inject printing, photolithography and chemical etching (Noblin et al., 2008; He et al., 2013; Koo and Velev, 2013). BPS molding was revolutionized with the introduction of PDMS for casting from microstructured templates more than 20 years ago (Effenhauser et al., 1997). PDMS is a commercially available silicon rubber, that is easy to use, optically transparent and biocompatible (Wu et al., 2016). Due to its many advantages, it is the most widely used material for soft lithography (Kamei et al., 2015; Chen et al., 2017). A simple two-step replica molding method for fabrication of BPS of plant leaves using PDMS whereby the microstructure was directly replicated from the leaf was introduced in 2014. Through this method the reliance for costly reagents and equipment such as inject printer or laser ablation was eliminated (Zhang et al., 2014). Here, we used a method similar to that presented by Zhang et al. (2014) however, we had to adjust it to tomato leaves, which have different trichomes than the ones on spinach leaves used by Zhang et al. (2014). The full description of this adjustment is presented in our previous publication (Alon et al., 2022). The leaf BPS platform was also used by us and others to study leaf-bacteria interactions in several systems, using different surfaces (Zhang et al., 2014; Doan and Leveau, 2015; Guttman et al., 2021; Gupta et al., 2022). However, this platform was rarely used to study this interaction with fungi. In addition to the BPS, as a control, we fabricated a PDMS surface without microstructure. Due to the chemical similarity of both surfaces, potential differences in fungal behavior could be interpreted as a result of thigmo-sensing of B. cinerea.

We observed different stages of the pre-penetration process. First, we found that the leaf microstructure influences the distribution of *B.* c*inerea* conidia. Most conidia were entrapped between cell depressions. In previous work, we have found that microstructure also influences the immediate attachment of *B. cinerea* conidia on synthetic surfaces (Alon et al., 2022). Immediate attachment is a passive process that includes even dead conidia (Doss et al., 1993). Therefore, immediate attachment cannot be considered as a thigmo-based response, because this is an active process that includes a signal pathway and a growth response (Almeida and Brand, 2017). Additionally, it is unclear whether the location of attachment would be different in the presence of epi-cuticular wax as observed in experiments with another pathogen - *S. nodorum* (Cunfer, 1984; Zelinger et al., 2006). However, pathogen/host relationship begins with immediate attachment, which is the base for the further infection process. This study provides evidence that microstructure influences the location of this attachment.

Second, we compared the germination of *B. cinerea* on flat and patterned PDMS in order to examine the impact of microstructure on this process. We found that the germination proportion of *B. cinerea* conidia was greater on a patterned surface than on a flat surface. This shows that, to some extent, the fungi recognized the surface of the PDMS replica as a tomato leaf, and therefore as a suitable host, which increased germination. However, germination proportion was still low (Nassr, 2013) and hence, other non-topographic factors, for example chemical stimuli, like the concentration of nutrients in the suspension or physical factors non-related to microstructure, clearly also play a role in germination initiation. The physical factors could be the surface hardness and hydrophobicity of the PDMS that was used for both samples (Doss et al., 1993; Doehlemann et al., 2006). We have also shown previously that the hydrophobicity of tomato leaf replica differs from that of tomato leaf, which, as mentioned, can play an important factor in *B. cinerea* behavior upon the surface (Alon et al., 2022).

Third, we compared the germ tube length of conidia incubated on patterned and flat surfaces. Differences could be observed between the groups, but they were not statistically significant. Development of invasion structure, characterized by the thickening of the germ tube in the context of appressoria formation appeared earlier and more frequently on leaf patterned PDMS. This suggests that the fungus recognized the PDMS replica as a potential host, because of its surface microstructure. Therefore, the observed development of invasion structure, which was induced by the surface topography, can be defined as thigmo-differentiation (Staples, 1983; Hoch et al., 1987). On the other hand, appressoria formation was also observed on flat PDMS. This shows that microstructure was not the only stimulus that induced development of invasion structure of *B. cinerea*. The nutrient in the suspension and the hydrophobicity of the surface might have been an additional stimulus (Doehlemann et al., 2006).

Directional growth towards stomata and along artificial ridges was shown for the fungus *Uromyces appendiculatus (* Hoch et al., 1987). However, in this work, the researchers were using polystyrene surface etched to form specific sized ridges. Using this technique, they tested only one structural feature. This is very useful, specifically for changing the properties of this feature; however, it does not incorporate the competition that might occur between several, important, structural features. In a subsequent work (Kwon and Hoch, 1991), the researchers also showed the dynamic of the process, which we also show here for *B. cinerea*, but under more diverse topographical conditions. Infectious structures were also shown to form in the wheat steam rust fungus *Puccinia graminis* f. sp. *tritici* in response to scratched plastic surfaces (Staples, 1983) which, again, did not mimic the exact microstructure of the leaf surface. A study by Wynn proceeded all above mentioned studies and did, in fact, use a synthetic leaf replica (Wynn, 1976). This study also used the bean rust fungus *Uromyces phaseoli* and showed that appressorium tends to form on stomatal lips in both natural leaves and synthetic replicas. In our system, directional growth could be observed for some germ tubes on the PDMS replica that seemingly grew along cell depressions. Our system used a necrotrophic fungus as opposed to previous systems that used biotrophic fungus. This could explain why all previous studies showed attraction to stomata while we observed almost no preference to stomata. This also shows the importance in using a whole leaf microstructural replica rather than specific structural elements in order to select for the most influential structural features. In the context of the classification of the results, it needs to be mentioned that *B. cinerea* is mainly transmitted through air currents and not through water droplets (Williamson et al., 2007), as simulated in the experiment. On the other hand, germination usually occurs in presence of moist leaf surfaces. Therefore, the experimental setting can be considered as a faithful imitation of the natural process during the initial stages of *B. cinerea* infection by conidia.

In conclusion, this study provides evidence that the pathogenic fungus *B. cinerea* has the ability to sense contact; even though this ability is only a part of a wide set of properties needed for a successful infection of the host, and chemical signals also play an important role (Doehlemann et al., 2006). The distribution of conidia according to structural features could be quantified, and we observed the tendency of conidia to become entrapped between cell depressions. Germination of *B. cinerea* conidia was shown to be a process partially influenced by leaf microstructure. Additionally, this study provides evidence for *B. cinerea* sense of touch, as contact induced directional growth (thigmotropism) of germ tubes and development of invasion structure (thigmo-differentiation) in accordance with topographical features could be observed. As previous research using synthetic replicas as a tool was performed more than 20 years ago, new tools in material sciences are now available and could be used in additional fungal systems. Further research, incorporating additional parameters combined with leaf microstructure in leaf-microorganism interaction of *B. cinerea* on tomato leaf would be desirable and would shed more light and further understanding towards reducing *B. cinerea* infections. Additionally, further research looking at the possibility of breeding for tolerance, based on microstructural features has the potential for applications concerning long term, structural based resistance which was not studied previously.

## Data availability statement

The original contributions presented in the study are included in the article/supplementary material. Further inquiries can be directed to the corresponding author.

## Author contributions

HR performed the experiments, analysed the data, and wrote the manuscript. HA assisted with statistical analysis. OS assisted with microscopy experiments and edited the manuscript. YE assisted with experiments and edited the manuscript. MK analysed the data, assisted with statistical analysis, wrote and edited the manuscript. All authors contributed to the article and approved the submitted version.

## Funding

This project has received funding from the Agriculture Chief Scientist in Israel, grant agreement no. 20-01-0200.

## Acknowledgments

We would like to acknowledge Dalia Rav-David for technical assistance and useful advice.

## Conflict of interest

The authors declare that the research was conducted in the absence of any commercial or financial relationships that could be construed as a potential conflict of interest.

## Publisher’s note

All claims expressed in this article are solely those of the authors and do not necessarily represent those of their affiliated organizations, or those of the publisher, the editors and the reviewers. Any product that may be evaluated in this article, or claim that may be made by its manufacturer, is not guaranteed or endorsed by the publisher.
